# Risk Perceptions, Knowledge and Behaviors of General and High-Risk Adult Populations Towards COVID-19: A Systematic Scoping Review

**DOI:** 10.3389/phrs.2021.1603979

**Published:** 2021-11-15

**Authors:** Nathalie Clavel, Janine Badr, Lara Gautier, Mélanie Lavoie-Tremblay, Jesseca Paquette

**Affiliations:** ^1^ Ingram School of Nursing, McGill University, Montreal, QC, Canada; ^2^ École de Santé Publique, Université de Montréal, Montreal, QC, Canada

**Keywords:** COVID-19, behavior, SARS-CoV-2, adult populations, knowledge, risk perceptions, high-risk adults

## Abstract

**Objectives:** The COVID-19 pandemic represents a major crisis for governments and populations. The public’s risk perceptions, knowledge, and behaviors are key factors that play a vital role in the transmission of infectious diseases. Our scoping review aims to map the early evidence on risk perceptions, knowledge, and behaviors of general and high-risk adult populations towards COVID-19.

**Methods:** A systematic scoping review was conducted of peer-reviewed articles in five databases on studies conducted during the early stages of COVID-19. Thirty-one studies meeting the inclusion criteria were appraised and analyzed.

**Results:** The levels of risk perceptions, knowledge, and behaviors towards COVID-19 were moderate to high in both general and high-risk adult populations. Adults were knowledgeable about preventive behaviors. Our review identified hand-washing and avoiding crowded places as dominant preventive behaviors. Being a female, older, more educated, and living in urban areas was associated with better knowledge of COVID-19 and appropriate preventive behaviors.

**Conclusion:** This review offers a first understanding of risk perceptions, knowledge and behaviors of adult populations during the early stages of the COVID-19 pandemic.

## Introduction

Coronavirus Disease 2019 (COVID-19) was declared a pandemic by the World Health Organization on March 11, 2020. Since then, COVID-19 continues to represent a major concern for populations and governments. As of early July 2021, more than 184 million cases have been confirmed and nearly 4 million confirmed COVID-19-related deaths have been reported globally [1, 2]. Among the general population, specific subgroups have been particularly affected by the pandemic, including older adults and individuals with underlying conditions who are at the greatest risk for developing severe complications [3]. Other vulnerable individuals such as those with lower socioeconomic status, and racial and ethnic groups have been hard hit by the virus, with an increased risk of getting sick and/or dying from COVID-19 [4–7].

Since the beginning of the COVID-19 pandemic, many studies have been conducted worldwide to understand people’s awareness and behavioral response towards the disease. Public risk perceptions (RPs), knowledge, and behaviors are key factors that play a vital role in the community transmission of infectious diseases [8, 9]. Studies conducted on previous coronavirus outbreaks and early research on COVID-19 transmission dynamics have shown that public awareness and compliance with preventive measures can have a significant impact on the trajectory of an outbreak [10–13].

Several behavior change models have been applied to assess public response to infectious outbreaks [14]. One of the most widely used in public health is the knowledge, attitude, and practice (KAP) model, which states that the adoption of a behavior in individuals is a step-wise process that first involves the acquisition of knowledge, and then the generation of good attitudes and appropriate practices [15]. Evidence has demonstrated that low KAP levels in individuals is associated with poor disease preventive behaviors [15, 16]. KAP studies conducted during past infectious disease outbreaks generally assessed various aspects of knowledge (e.g., routes of transmission, common symptoms, preventive behaviors), attitudes (e.g., risk perceptions, impact on daily life) and preventive practices (avoidance behaviors, mask wearing, social distancing) [10, 11, 17]. The health belief model, another widely used health-related behavioral model, argues that RPs, including perceived susceptibility and perceived severity of a disease, are key contributors to people’s behavior changes during pandemics [14]. Evidence indicates that higher perceived risk of infection is associated with increased adoption of preventive measures against infection [14, 18]. Simultaneously identifying RPs, knowledge, and behaviors (RPKB) of general adult populations and high-risk adults can inform risk communication strategies and interventions to better control the spread of COVID-19.

While all populations are affected by COVID-19 worldwide, high-risk and vulnerable individuals are facing a disproportionate burden of cases and deaths. Previous studies have shown that sociodemographic patterns can play a role in individuals’ perceptions and behaviors with regards to an infectious disease [19, 20]. Current data shows important disparities in COVID-19 cases and deaths particularly among lower socioeconomic, racial, and ethnic groups of the population [7, 21]. As the virus does not affect individuals equally, the factors associated with RPKB that can explain why certain population groups are more likely to get infected with COVID-19 must be identified. Knowing the predictors of RPKB can help mitigate the negative effects of COVID-19 in high-risk groups as more targeted strategies can be developed to facilitate engagement in the preventive measures.

To the best of our knowledge, no overviews have been published of primary studies assessing RPKB of general adult populations and high-risk or vulnerable adults with regards to COVID-19. The objectives of our scoping review were therefore to 1) conduct a systematic search of the recently published primary studies assessing RPKB of the general adult population and high-risk adults with regards to COVID-19; 2) map the characteristics of the identified studies; 3) identify the levels of RPKB towards COVID-19 in both adult general populations and high-risk adults; and 4) understand the factors associated with RPKB.

## Methods

Given the high number of studies conducted with only short delays, we decided to conduct a scoping review to map the early evidence regarding our research questions [22]. To ensure a systematic approach in conducting our scoping review and for reporting the findings, we followed the PRISMA extension for scoping reviews (PRISMA-ScR) [23]. Before conducting the review, we published a research protocol on the protocols IO research platform [24]. We conducted a comprehensive search of the following electronic databases: MEDLINE-Ovid, EMBASE-Ovid, PsycINFO-Ovid, Web of Science, and CINAHL (EBSCO). The searches were performed in English, with the search terms in Table 1.

**TABLE 1:** Search terms (COVID-19 scoping review project, Canada, 2020–2021).

Population	Perception	Knowledge	Behavior
Public	Perception	Knowledge	Behavior
	Risk perception		
People	Awareness	Comprehension	Practice
Person	Consciousness		Action
Individual			
Resident			
Citizen			
Adult			
Community			
Group			
Patient			

The search term strategies were developed with support from a librarian at McGill University. Our search strategy for MEDLINE-Ovid is shown in Supplementary Appendix S1. Searches using the four other databases are available upon request. A comprehensive search of the gray literature was undertaken through Open Grey, Scopus, Wonder, Social Science Research Network, and MedRxiv. We also searched the World Health Organization, Centers for Disease Control and Prevention, European Centre for Disease Prevention and Control, and the Center for Infectious Disease Research and Policy websites.

### Inclusion and Exclusion Criteria

We included peer-reviewed and preprint articles that assessed RPKB of general adult populations or high-risk adults with regards to COVID-19. High-risk groups were defined based on the Centers for Disease Control and Prevention’s definition [3]. CDC has defined groups of individuals at increased risk of developing severe illness from SARS-CoV-2 (including older adults, people with medical conditions, and pregnant women) and other groups of adults who need to take extra precautions because of their higher risk of getting infected (including racial and ethnic racial groups, people with disabilities, the homeless, refugee populations, etc.) [3].

We decided to exclude studies that did not simultaneously assess the three determinants of the COVID-19 transmission dynamics analyzed in our scoping review (i.e., risk perceptions, knowledge, and behaviors) because we wanted to compare the studies based on each of these three factors that all play a role in the transmission of the virus. The inclusion and exclusion criteria are shown in Table 2.

**TABLE 2:** Inclusion and exclusion criteria (COVID-19 scoping review project, Canada, 2020–2021).

Inclusion criteria	Exclusion criteria
Peer reviewed or preprint articles	Studies not based on original research (e.g., editorial, opinion, or commentary papers)
Risk perceptions, knowledge and behaviors towards COVID-19	Studies that did not simultaneously assess risk perceptions, knowledge, and behaviors towards COVID-19
Adults	Children or adolescents
General or high-risk populations	Healthcare workers or students in medicine, dentistry or health sciences (e.g., nursing)
Any study design	Studies using data from online posts or searches (e.g., data from Google searches)
English language	
Published or posted between January and August 2020	

### Data Screening and Extraction

Three authors (NC, JB and JP) independently reviewed the titles and abstracts of the articles against our inclusion and exclusion criteria. A pilot round with a randomly generated sample of nearly 10% of the articles was done to evaluate inter-reviewer agreement on the exclusion and inclusion criteria before a full screening was done for all articles [25, 26]. After the initial review of full-text articles, five authors (NC, JB, LG, MLT and JP) completed the data extraction. The data extraction consisted of collecting variables on the 1) general characteristics of the articles (i.e., authors, title, month of publication, country, and publication journal); 2) characteristics of the participants (i.e., data collection period and method, targeted population, sample size and characteristics, sampling scheme, response rate, and statistical analysis); and 3) research question outcomes (i.e., RPKB in general and high-risk adult populations and their associated factors).

### Quality Appraisal

In the context of the COVID-19 pandemic where studies have been conducted in very short time-frames, we decided to assess the quality of the included articles. Since all articles were cross-sectional studies, the quality of studies was assessed using the appraisal tool for cross-sectional studies (AXIS tool) [27], which was developed to appraise observational cross-sectional studies [27]. Quality appraisal was conducted independently by the four co-authors (NC, JB, LG, MLT and JP). Discrepancies were resolved through discussions between the first author (NC) and the co-authors (JB, LG, MLT and JP). A total score was assigned to each article, which was then grouped into one of three categories: 1) low quality (<60%), 2) medium quality (60–80%), and 3) high quality (≥80%). Low quality studies were not excluded from the scoping review.

## Results

**FIGURE 1:**
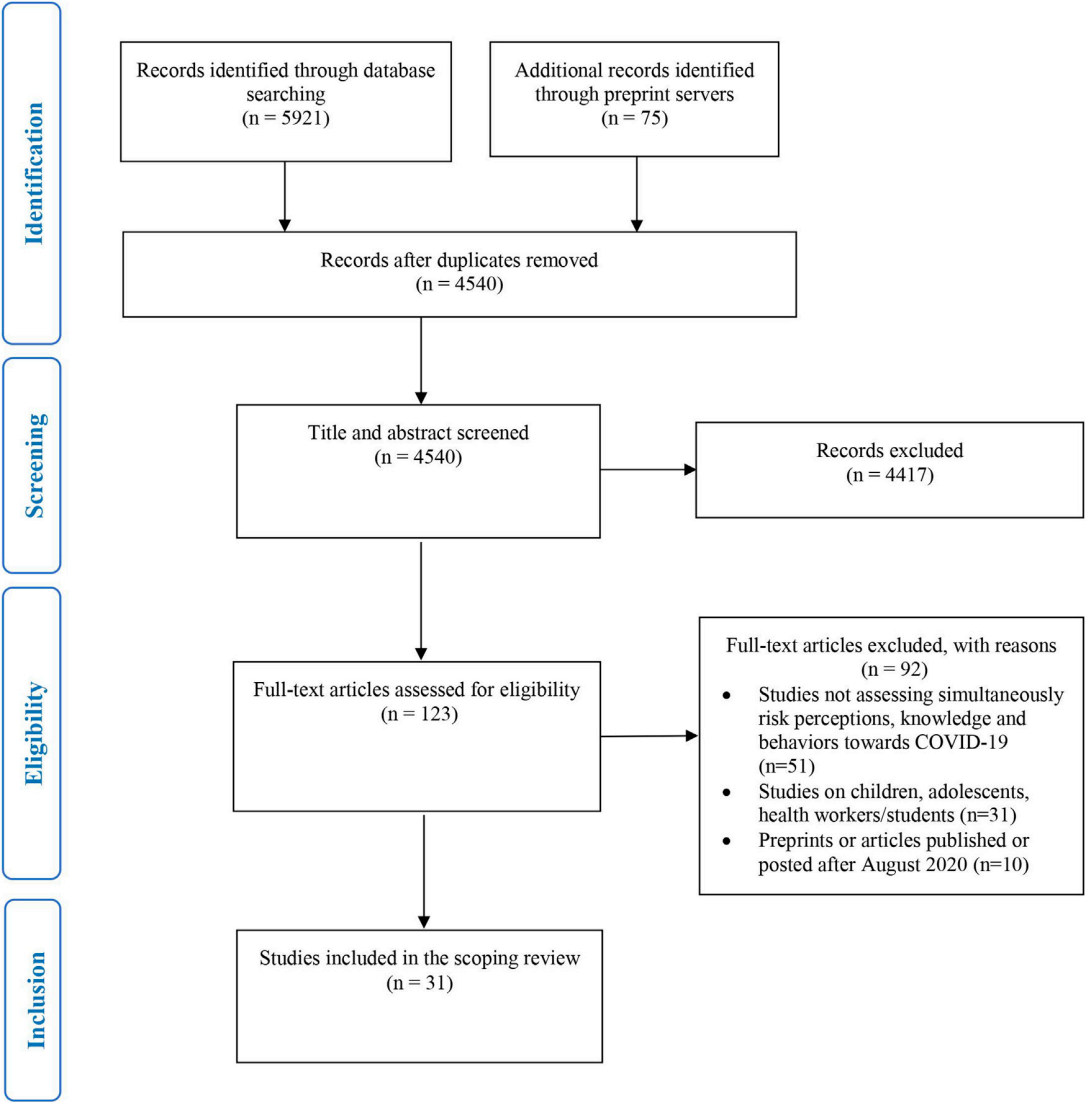
PRISMA flow diagram.

Our initial search yielded 5,921 articles and 75 additional records were identified through the preprint servers. After removing duplicates and reviewing the titles and abstracts, 31 articles met our inclusion criteria (see the PRISMA flow diagram in Figure 1).

### General Characteristics of the Included Studies

All of the studies were cross-sectional, using surveys conducted between January and May 2020. Most of the studies (84%) were based on non-probability sampling methods, including convenience samples (52%), snowball sampling methods (19%), or quota samples (13%). Only 16% of the studies used simple or stratified random sampling methods. A total of 51% of the studies had samples that were smaller than 1,000 participants. Finally, 87% of the articles used both descriptive and advanced statistics to present the survey data and analyze factors that were significantly associated with RPKB towards COVID-19. The main characteristics of the included studies are shown in Table 3.

**TABLE 3:** General characteristics of the included studies (*n* = 31) (COVID-19 scoping review project, Canada, 2020–2021).

	Number (%) of studies
Study design	
Cross-sectional (surveys)	31 (100)
Sample size	
<500	5 (16)
≥500–999	11 (35)
≥1000–1999	7 (23)
≥2000	8 (26)
Sampling scheme	
Convenience sampling	16 (52)
Random and stratified sampling	5 (16)
Snowball sampling	6 (19)
Quota sampling	4 (13)
Data collection period*	
January	1 (3)
February	6 (19)
March	18 (58)
April	9 (29)
May	5 (16)
Use or adaptation of an existing scale/survey	
Yes	9 (29)
No	22 (71)
Data collection method/mode of administration**	
Online survey (self-administered)	21 (68)
Phone or face-to-face (administered by an interviewer)	10 (32)
Paper survey (self-administered)	2 (6)
Statistical analysis	
Descriptive statistics (only)	4 (13)
Descriptive and advanced statistics (analysis of variance/regression analysis)	27 (87)

*The total exceeds 100% because several studies collected data during two consecutive months.

**The total sometimes exceeds 100%, because two studies used two survey administration methods for the participants.

### Characteristics of the Participants

The survey participants were from 21 countries across 5 continents (Africa, Asia, Europe, North America, and Oceania). Most of the studies assessed RPKB towards COVID-19 among general adult populations (*n* = 20), while 11 studies focused on high-risk adults. All studies collected demographic statistics on age and most collected data on gender and level of education. Other data were related to living areas, occupation, health status, and ethnicity. More detailed information is provided in Table 4 and Supplementary Appendix S2.

**TABLE 4:** Characteristics of the participants (COVID-19 scoping review project, Canada, 2020–2021).

	Number (%) of studies
Country of residence	
India	5 (16)
United States	4 (13)
Turkey	3 (10)
Germany	2 (6)
Hong Kong	2 (6)
Australia	1 (3)
Bangladesh	1 (3)
Canada	1 (3)
China	1 (3)
Egypt	1 (3)
Ethiopia	1 (3)
Italy	1 (3)
Kenya	1 (3)
Korea	1 (3)
Malawi	1 (3)
Philippines	1 (3)
Serbia	1 (3)
Sudan	1 (3)
Taiwan	1 (3)
Uganda	1 (3)
United Kingdom	1 (3)
Population targeted	
General adult population	20 (65)
Adults with a chronic condition	6 (20)
Poor households	2 (6)
Elderly persons	1 (3)
Pregnant women	1 (3)
Sexual minorities	1 (3)
Demographic and other statistics collected*	
Age	31 (100)
Gender	30 (97)
Level of education	26 (84)
Income	15 (48)
Living areas	17 (55)
Occupation	13 (42)
Health status or chronic condition	9 (29)
Ethnicity	6 (19)

*The total sometimes exceeds 100%, because some studies collected different types of statistics for the participants.

### Quality Appraisal of the Included Studies

Overall, the quality of the included studies was moderate. The mean score calculated using the AXIS quality appraisal tool was 64%. Most studies were of medium quality (*n* = 16), ten were of low quality and five studies were of high quality. Low-quality articles were mostly of studies conducted in general adult populations that used online surveys and convenience sampling or snowball methods to recruit participants without accounting for possible selection and non-response bias, which led to sampling bias with young or educated participants being overrepresented in the samples. These studies usually did not report the response rate and did not provide any information about non-responders, which can lead to non-response bias and over- or under-representation of certain categories of the populations. Finally, most of the articles (*n* = 22) did not report using or adapting existing tools to assess RPKB related to COVID-19. The detailed quality appraisal grid is shown in Supplementary Appendix S3. The moderate quality of the included articles implies that the results should be interpreted carefully, especially for the lowest-rated studies.

### RPKB in General and High-Risk Adult Populations

#### Risk Perceptions Towards COVID-19

In 80% of the studies (*n* = 25), RPs towards COVID-19 were assessed through perceived susceptibility for self and/or others to be infected. Only one study also measured risk perception by considering the concept of the perceived risk of infecting others [28, 29]. Perceived severity of COVID-19 in the community or for high-risk groups was assessed in 65% of the studies (*n* = 20). RPs were reported by the percentage of participants who reported being worried about getting infected or by calculating the mean score of the perceived likelihood of becoming infected (low or high scores).

#### Perceived Susceptibility Towards COVID-19

##### General Adult Populations

Overall, the studies found that perceived susceptibility for self was moderate in the populations from countries situated in the five continents (Africa, Asia, Europe, North America and Oceania). Seven studies conducted in Canada [29] China [30], Hong Kong [31], India [32, 33], Malawi [34] and the United States [35] reported a moderate proportion of adults being worried about getting infected. The percentage of respondents who were worried about getting infected with COVID-19 ranged from 40% [32] to 67% [31]. In the study conducted in Canada, respondents reported being more concerned about a family member contracting COVID-19 than about contracting the virus themselves [29]. Only two studies in Egypt [36] and India [37] showed a higher proportion of participants (87 and 82% respectively) being worried about getting infected. Among studies that measured the perceived likelihood of getting sick from COVID-19 among adults in Australia [38], Italy [39], South Korea [40] and Serbia [41] and the United States [42], the perceived risk was moderate. The mean scores of perceived susceptibility ranged from 2/5 [42] to 3.8/5 [39].

##### High-Risk Adults

Most of the studies conducted among high-risk adults also found a high level of perceived susceptibility towards COVID-19. Five studies reported a high percentage of participants being worried about getting infected (ranging from 64 to 88%). These studies were conducted among adults living with a chronic disease in the United States [43] and India [44], poor households in the Philippines [45], sexual minorities in Taiwan [46] or liver transplantation recipients and candidates for transplants in Germany [47]. In addition, two studies reported a low percentage of perceived susceptibility among the participants (35%); one was conducted among pregnant women in Turkey [48] and the other was in poor households in Kenya [49].

#### Perceived Severity of COVID-19

##### General Adult Populations

The perceived severity of COVID-19 among the participants was high in most studies that measured this variable (*n* = 10). The proportion of participants who perceived COVID-19 as a threat to their health ranged from 70 to 97%, in studies in Australia [38], Hong Kong [31, 50], India [33], Malawi [34], Sudan [51], and Uganda [52]. Interestingly, the comparative study conducted in two countries showed that respondents from Hong-Kong (97%) were far more worried about having complications compared to the respondents from the United Kingdom (21%). We noted the exception of one study that was conducted in Bangladesh where 55% of the participants considered COVID-19 as a deadly disease [53]. In studies that measured perceived severity, the mean scores among participants in South Korea [40], Serbia [41] and Turkey [54] ranged from 3.66/5 to 4.7/5 [40, 41].

##### High-Risk Adults

As in the general adult populations, the perceived severity of the disease among high-risk adults was significant. Six studies reported a high proportion of participants who perceived COVID-19 (68–95%) as a serious threat for themselves. The studies were conducted among elderly persons [30], liver recipients and candidates for transplants [47], adults with Parkinson’s Disease [55], young adults with Type 1 diabetes [44] and sexual minorities [46], and poor households [49]. In the study conducted among pregnant women, only 51% of the women felt more vulnerable to developing complications from COVID-19 [48].

#### Knowledge of COVID-19

Knowledge related to COVID-19 was mainly assessed through five main variables: modes of transmission (*n* = 20), common symptoms (*n* = 14), perceived general level of knowledge (*n* = 14), preventive behaviors to avoid infection (*n* = 12), and high-risk groups (*n* = 6). The level of knowledge for the different variables was calculated using scores (high or low scores) or only reported in the percentage of participants. Several studies reported only an overall knowledge score or a proportion of respondents with a high level of knowledge (generally at least 70% correct answers), without reporting scores for each knowledge variable measured (e.g., symptoms, modes of transmission) [35, 52, 53, 56].

##### General Adult Populations

Adult participants had an overall good knowledge of COVID-19. In most studies, the overall knowledge rates and the proportions of knowledgeable respondents were relatively high (71–98% of respondents) [28, 36, 39, 40, 51, 52, 56]. In three studies, however, the survey results showed that respondents were not very knowledgeable: a mean score of 8.56/13 among Bangladeshi respondents [53] and 41 and 50% of respondents having poor knowledge among the adults in the United States [35] and Malawi [34], respectively. Finally, one study showed a very important knowledge gap since 64% of respondents had never heard about COVID-19 in Turkey [54]. In addition, three studies in Hong Kong [31], India [37], and Sudan [51] found that a significant proportion of individuals (ranging from 24 to 56%) were not aware that asymptomatic persons can infect others/or know the period of asymptomatic incubation. In contrast, the Canadian study showed that 86% of the respondents knew about asymptomatic transmission [29]. Similarly, a very high proportion of respondents (>90%) identified that the disease could be transmitted through droplets, and direct or indirect contact (from 71 to 99%) [29, 31, 33, 36, 38, 39, 42, 50], except in two studies where only 60% of the Malawi participants and 29% of the Indian respondents knew that COVID-19 spreads through multiple modes like touching, kissing, and sneezing [32, 34]. Adult respondents were also very knowledgeable about the common symptoms of COVID-19. In several studies, a minimum of 80% of the respondents (up to 98%) knew all of the common symptoms of COVID-19 [36, 38, 41, 42, 51], except in one study conducted among Indian adults where only 18% of the respondents considered fever to be a symptom of the disease [32]. Finally, adult participants were very knowledgeable about preventive practices to avoid COVID-19 transmission, with more than 75% of the respondents acknowledging the preventive behaviors like social distancing, hand washing/sanitizing, wearing a mask, and avoiding public gatherings [32, 36–39].

##### High-Risk Adults

Studies focusing on high-risk adults also found that most respondents were very knowledgeable about COVID-19, including common symptoms, routes of transmission, and behaviors to avoid an infection (proportions of respondents were between 77 and 94%) [30, 44–47, 49, 57]. As for adults in general populations, two studies found that only 56% of adults with chronic diseases [58] and 26% of liver recipients and candidates for transplants thought that COVID-19 can be spread by asymptomatic patients [57]. The two studies conducted with poor households also showed a lack of knowledge about difficulty in breathing being a common symptom of COVID-19 [49] and some preventive behaviors such as social distancing, wearing a mask, and avoiding crowded places [45]. Finally, the study conducted with pregnant women revealed an important lack of knowledge regarding the impact of COVID-19 on preterm births [48].

#### Preventive Behaviors Towards COVID-19

Several preventive behaviors were assessed in the 31 included studies, including hand-washing (*n* = 24), wearing a mask (*n* = 23), avoidance behaviors (e.g., avoiding crowded places, social gatherings or public transports, cancelling travel) (*n* = 18), staying at home/reducing social contacts (*n* = 14), and practicing social distancing (*n* = 12).

##### General Adult Populations

In studies on general populations, many authors reported appropriate behaviors for preventing COVID-19. The most observed preventive behavior was washing hands frequently, with reported rates from 68 to 99% among respondents [31–34, 36–38, 40–42, 50–52, 54]. According to several studies, avoiding crowded places or social gatherings were also practices generally adopted by most respondents (from 59 to 99%) [32, 37, 51–53], except in two studies where a minority of South Korean participants (41%) [40] and half of the Malawian respondents [34] reported avoiding crowded places. In any case, adults were reportedly more or less compliant when it comes to staying at home, reducing social contacts, or avoiding public transport. In three studies, more than 80% of respondents followed these practices [28, 32, 42, 52] whereas studies conducted among Hong Kong and South Korean adults showed lower rates in adopting preventive behaviors; 53 and 39% of the respondents, respectively, reported avoiding public transport [31, 40]. Mask wearing was also variably followed in the studies. While five studies reported high proportions of respondents (63–97%) wearing masks [31, 33, 37, 40, 52–54, 56], four other studies conducted among Egyptian, Serbian, Sudanese, and Indian adults showed much lower compliance (around 35%) [32, 36, 38, 41, 51]. The findings of the comparative study highlighted a huge difference between Hong Kong respondents (97%) and participants from the United Kingdom (3%) in wearing masks [50]. Finally, practicing social distancing was also variably followed. For example, studies conducted among Canadians, Ugandans and Serbians showed more compliance among the participants with this practice [29, 41, 52] compared to the respondents in South Korea [40].

##### High-Risk Adults

Overall, studies on the high-risk adults have reported appropriate preventive behaviors during the early periods of the pandemic. Three studies reported that a large majority of respondents (>90%) among adults with type 1 diabetes, poor households, liver recipients, and candidates for transplants washed their hands more frequently [44, 47, 49]. Nevertheless, only 40% of the adults with Parkinson’s disease reported washing their hands more frequently [55]. Staying at home or leaving home less frequently were also reported by a majority of respondents (60–79%) from poor households [49], liver transplant recipients, candidates for transplants [47, 57], and people with Parkinson’s disease [55]. Most of the respondents (63–94%) from poor households [45, 49], elderly persons [30] and sexual minorities [46] avoided crowded places or stopped attending social gatherings. In four studies involving liver transplantation recipients and candidates for transplants in India [57], poor households [45], adults with chronic diseases in Ethiopia [58] and adults with Parkinson’s disease [55] few participants reported wearing a mask (6–37%). Nevertheless, in three studies with chronic patients and older adults, a higher proportion of respondents reported wearing a mask when leaving home [30, 44, 47]. Finally, three studies conducted among adults with type 1 diabetes [44], poor households in Kenya [49] and the Philippines [45] reported a high proportion of respondents (95 and 66%, respectively) keeping distance from other people to avoid getting infected with COVID-19.

#### Factors Associated With RPKB Towards COVID-19

The most studied factors that were significantly associated with RPKB towards COVID-19 were socio-demographic factors such as age, gender, education, ethnicity, and living areas.

##### Age

Studies in Hong Kong [31] and South Korea [40] found that older adults were significantly less worried about getting infected compared to the young adults. We found one exception to this in a study among poor households in Kenya where the perception of risk increased by age group [49]. Age was positively associated with perceived severity in three studies: one in Serbia among the public [41] and two other studies in the United States among the general public [56] and persons with chronic conditions [43]. In five studies, older adults were also found to be more knowledgeable about COVID-19 compared to younger adults [41, 42, 47, 53, 58]. In addition, five studies found that age was positively associated with hand-washing and other protective behaviors [30, 38, 40, 42, 51]. The authors of the study conducted in China highlighted that the Chinese elderly held an ethical duty to protect others, which facilitated adherence to precautionary measures [30].

##### Gender

In two studies, women were significantly more worried about being infected with SARS-CoV-2 or to consider COVID-19 as a threat to health [43, 47, 55]. Men were found to be less knowledgeable than women about COVID-19 in three studies [41, 42, 51]. Finally, a high number of studies reported that the adoption of preventive behaviors such as hand-washing, wearing a mask, reducing contacts, avoiding social gatherings, or practicing social distancing was positively associated with the female gender [28, 31, 35, 38, 40, 42, 43, 50, 51, 53].

##### Level of Education

A higher level of education was positively associated with knowledge of COVID-19 in seven studies, especially with regards to modes of transmission and common symptoms [31, 41, 44, 45, 49, 51, 58]. Adults who were educated were also more likely to adopt preventive behaviors like wearing a mask or maintaining social distance [30, 40, 41, 45].

##### Ethnicity

Significant differences were found in RPs between ethnic groups in three studies that reported that Black respondents were less likely to be worried about getting COVID-19 [35, 43, 56]. In three studies, the Black respondents were also less likely than the White respondents to have a high knowledge of COVID-19 [35, 42, 43]. Finally, some studies showed mixed evidence regarding ethnic disparities in adopting preventive behaviors. One study with a low-quality rating was conducted in the general population. In this study, Black people were reported to be more likely to have good practices towards the transmission of COVID-19 [35]. Two other studies, with a medium-quality rating, involving patients with chronic conditions and the general population showed the opposite results [43, 59]. One study conducted in the general population reported that African Americans were more likely to leave their homes. The authors suggested that the differences observed might be related to social circumstances since African Americans are more likely to work in the public sector, and hence, they are less likely to work remotely [59]. Finally, the study conducted in Australia showed that non-Caucasian residents (Asian and Australian aboriginal) reported more protective behaviors than Caucasian respondents [38].

##### Living Areas

Studies in Canada and Malawi found that people living in rural or less populated areas were less likely to worry about contracting the virus [29, 34]. The study conducted in Italy showed that the concerns of contracting SARS-CoV-2 increased with geographic proximity to the center of the outbreak, and that people living in COVID-19 hotspot zones were more likely to worry about getting infected [39]. Three studies showed that rural residents living in Ethiopia, Malawi, and India were also less knowledgeable than people living in urban areas [34, 44, 58]. These studies highlighted the fact that people living in urban communities tend to have better access to information and awareness campaigns that are conveyed by the social, digital, or print media. Finally, rural residents in Ethiopia, Malawi, and China reported poorer preventive practices than urban residents [30, 34, 58] and people living in COVID-19 hotspot zones in Italy were more likely to adopt precautionary behaviors than people living in less affected areas [39].

## Discussion

To the best of our knowledge, this is the first scoping review that offers a mapping of studies conducted among general and high-risk adult populations on RPKB towards COVID-19 and factors associated with RPKB.

Our scoping review has several limitations. We decided to cover a short period (January to August 2020). In the context of the pandemic, many studies were conducted and published very early. Consequently, a first step in mapping the emerging evidence has been to gain an understanding of RPKB towards COVID-19 in the early stages of the pandemic. We did not provide a cross-comparison between countries, mainly because of the significant heterogeneity of studies regarding survey and sampling methods and the absence of cross-country studies in the scoping review. Finally, because the overall quality of the included articles was moderate, the research findings presented here should be interpreted carefully. Most studies used non-probability sampling and online surveys that raise doubts about the capacity for the authors to generalize the research findings. While online surveys allow for rapid and user-friendly data collection from large samples of the population, they can also increase the likelihood of sampling and non-response bias [60].

Overall, in the early months of the pandemic, the levels of RPKB towards COVID-19 were moderate to high in both general and high-risk populations. We did not notice significant differences in RPs between the general and the high-risk adult populations. Nevertheless, two studies, one with pregnant women in Turkey [48] and the other with poor households in Kenya [49] reported low-risk perception levels, in contrast to six other studies conducted among high-risk adults [43, 45–47, 55, 57]. Interestingly, overall, the perceived severity of the disease was slightly higher than the perceived susceptibility of getting COVID-19 during the first months of the pandemic. Similar findings were reported in an international study by Zwart et al. [61] on RPs related to SARS-CoV, which revealed an intermediate level of SARS vulnerability and a high perceived severity, in comparison to other diseases [61]. This finding might seem counterintuitive since we now know that the case-fatality rates for COVID-19 are relatively low while the transmissibility rates are high, in comparison to other coronavirus disease outbreaks [62]. Nevertheless, several explanations could be given for this finding. During the first wave of the COVID-19 pandemic, a delay occurred before a scientific consensus on mortality rates and asymptomatic transmission emerged and before the public was informed. Another explanation could be that during the first months of the pandemic, cases of COVID-19 were highly concentrated in certain regions (e.g., Hubei in China, Lombardy in Italy) or cities (e.g., Wuhan, Milan, New York City) [63, 64], which providing a false sense of security towards COVID-19 transmission for people living outside the COVID-19 hotspots. Finally, the anxiety caused by the media and the memory of past fatal outbreaks, such as those caused by MERS and Ebola could explain that people were more worried about dying from the disease than being infected at the beginning of the pandemic. This explanation is in line with Zwart et al.’s findings on RPs during the SARS outbreak that indicated that more unfamiliar diseases can be perceived as being more severe [61].

The scoping review showed that general and high-risk adults were knowledgeable about COVID-19. Our findings are consistent with those of Majid et al.’s recent scoping review on knowledge, RPs, and behavior change during pandemics, where the authors stated that knowledge generally spreads rapidly during pandemics in most regions [65]. Nevertheless, we found exceptions in several studies that reported a relatively low level of overall knowledge among the general public in Bangladesh [53], and the United States [35]. Overall, the participants were very knowledgeable about preventive behaviors, including hand-washing, mask-wearing, social distancing, and avoidance behaviors. Nevertheless, an important knowledge gap on the asymptomatic transmission of COVID-19 was reported in many studies [31, 37, 51] as the asymptomatic nature of the virus transmission had not been clearly scientifically identified or shared with the public during the first wave of the pandemic. A high proportion of the respondents from poor households in Kenya did not identify difficulty in breathing as one of the main symptoms of COVID-19 [49]. Similarly, the pregnant women participants in Turkey were unaware that COVID-19 can cause preterm births [48], though this study was evaluated to be of low quality. Conversely, Maharlouei et al. [66] found that a greater proportion of pregnant women were aware of the risk of severe complications during birth.

Our review identified hand-washing and avoiding crowded places as dominant preventive behaviors among both general and high-risk adults at the early stages of the pandemic. Nevertheless, staying at home, reducing social contacts, and avoiding public transport were less widespread in general populations. Alternatively, the high-risk adults reported being much more compliant with staying at home or leaving home less frequently [30, 44, 47, 49, 57]. Wearing a mask was the least respected practice in the early stages of the pandemic for both general and high-risk adults, except in six studies that reported a high level of compliance with mask wearing [30, 31, 33, 50, 56, 67]. In Majid et al.’s scoping review, the authors reported varying degrees of adopting mask wearing, ranging from 4% in the United States to 96% in China [65]. In East Asia, mask wearing is socially embedded as a general preventive practice [68]. Surprisingly, a large majority of the participants from poor households [45, 49], including those living in slums in Kenya [49] reported using social distancing to avoid getting infected. This finding is in contradiction with a recent observational study conducted in an urban slum in India. In any case, the authors concluded that social distancing measures were more of an aspiration than reality [69].

Our review highlighted the existence of significant sociodemographic differences in RPKB towards COVID-19. Being a female, older, more educated and living in urban areas/or in hotspot zones was associated with higher risk perceptions, better knowledge about COVID-19 and appropriate preventive behaviors. This finding is consistent with Bish and Michie’s review on the determinants of protective behaviors during a pandemic [9]. We also found that age was negatively associated with perceived susceptibility to and positively associated with perceived severity of the disease. In a recent study on the age effect on preventive behaviors during the COVID-19 pandemic, the authors concluded that the oldest adults underestimated the probability of getting infected but at the same time they were aware of the COVID-19 threat [70]. Several arguments have been highlighted to explain gender differences in risk perceptions and preventive behaviors towards infectious diseases. Several studies have reported the women’s greater psychological vulnerability that can explain the increased worry about infectious outbreaks [71, 72]. Other evidence highlights the fact that women might feel more responsible for themselves and their families because of their greater involvement in caregiving activities [73, 74]. Finally, one study argues that women’s higher trust in government actions may result in higher risk perception regarding COVID-19 [75]. Educational-related differences in RPKB might be explained by a lower level of literacy among less-educated individuals that may comprise their understanding of the COVID-19 transmission and protective behaviors [76]. Other studies suggest that individuals with lower levels of education rely more on social media and less on public health resources or other official and reliable sources of information to gain information on COVID-19, which may lead to misinformation and inappropriate behaviors [77, 78]. From this review we also found that adults living in urban areas or hotspot zones were more likely to worry about getting infected, more knowledgeable, and more likely to adopt preventive practices [29, 30, 34, 39, 44, 58]. This difference in risk perception between hotspots and safe-zones agrees with a study conducted in China during the early stage of COVID-19. Shanghai respondents were reported to have significantly lower perceived susceptibility and higher severity, compared to their counterparts in Wuhan [79]. One recent study has also shown that people living in urban areas, which are often the main sites of infection because of their high population density [80], were more aware of the risk of infections and more likely to adopt preventive behaviors [81].

We also found several studies that showed Black respondents being less worried about getting infected and less knowledgeable about COVID-19, compared to White respondents [35, 42, 43, 56]. The history of racism has left Black communities with fewer educational and economic opportunities than their White counterparts and exposed them to higher social and health risks with important negative effects. In the context of the COVID-19 pandemic, structural factors such as lower education and income levels and limited access to public health information resources among Black communities may have explained why Black people were less knowledgeable and consequently less worried about COVID-19. At first glance, we might hypothesize that lower RPs and knowledge about COVID-19 among Black individuals might lead them to be less compliant with preventive behaviors. Nevertheless, mixed evidence has been given for the link between ethnicity and adoption of preventive behaviors, a finding that is consistent with the review of Bish and Michie [9].

### Conclusion and Future Research

Our findings have several implications for public health authorities aiming to be responsive in adopting appropriate and effective risk communication strategies at the very early stages of a pandemic. Our review shows that during the early stages of COVID-19, the perceived severity of COVID-19 was higher than the perceived susceptibility among general and high-risk adult populations. This finding suggests that people, especially those in less-affected areas, might have underestimated the infectivity of the virus. Perceived susceptibility combined with perceived severity plays a vital role in motivating health protection behaviors [82] and may facilitate or reduce the transmission of a virus during pandemics [14, 18]. While many countries have faced a third wave of COVID-19, RPs should be continuously monitored to adjust the risk communication strategies over time. Effective risk communication relies on generating a sense of worry among the public while avoiding the fear that could lead to denial and inappropriate behaviors [83]. Communication strategies must target certain groups of the population, including men, young and less educated adults who are less likely to perceive the risks associated with COVID-19, and those who are less knowledgeable and less likely to adopt preventive behaviors. Addressing literacy and numeracy issues in less-educated people can be achieved by delivering simple communications using videos about the preventive behaviors [84]. Young adults can be targeted by using accessible, credible, and reliable social media channels for providing information about COVID-19 [85].

While this review offers an initial understanding of RPKB of adult populations during the early stages of the COVID-19 pandemic, further research is needed to assess the psychological and behavioral responses over time. Whether people from ethnic minorities are less or more likely to have high levels of preventive behaviors towards COVID-19 is unknown. In any case, the disproportionate number of COVID-19 fatalities within the Black population [4, 6] should alert us to possible gaps in RPKB towards COVID-19 in these communities. Other vulnerable populations paid a heavy price because of the COVID-19 pandemic, like immigrant and refugees who live in precarious material and economic conditions [86]. Additional studies on health inequities experienced by marginalized populations, including ethnic minorities, immigrant and refugees may help public health authorities to introduce targeted actions towards these communities during the COVID-19 pandemic. While population-based surveys allow for a rapid assessment of psychological and behavioral responses of populations, in-depth qualitative studies are necessary to acquire a deeper understanding of RPKB towards COVID-19 among high-risk groups in the population.
