# Supplementary material for: Risk Perceptions, Knowledge and Behaviors of General and High-Risk Adult Populations Towards COVID-19: A Systematic Scoping Review
Source: Public Health Rev. 2021 Nov 15;42:1603979. doi: 10.3389/phrs.2021.1603979 (PMC8636827; doi:10.3389/phrs.2021.1603979)
Supplement: Supplementary file 1 [file DataSheet2.pdf]

Appendix 2 – Overview of the main characteristics and findings of the studies included in the scoping review.

Authors	Title	Country	Target population	Survey administration	Sampling scheme	Sample size	Sample characteristics	Main findings
Abdelhafiz AS, Mohammed Z, et al.	Knowledge, Perceptions, and Attitude of Egyptians Towards the Novel Coronavirus Disease (COVID-19)	Egypt	Adults living in Egypt	Online survey and in-person interview	Convenience	559	62% female, 38% male<br><br>18-30 (48%)<br>30-50 (39%)<br>50-60 (8.8%)<br>≥60 (4.1%)<br><br>Primary (2.1%)<br>High School (25.2%)<br>University higher studies (69.9%)	86,9% concerned of being infected or a family member.<br><br>Mean knowledge score was 16.39/23<br><br>Knowledge was lower among older, rural, less educated, and lower income groups.<br><br>The majority adopted appropriate preventive behaviors
Akula Y, Ayelign B, et al.	Knowledge, Attitude and Practice Towards COVID-19 Among Chronic Disease Patients at Addis Zemen Hospital, Northwest Ethiopia	Ethiopia	Adults living in Northwest Ethiopia	In-person	Convenience	404	39% women, 61% men<br><br>Age: 56.5± 13.5<br><br>Unable to read & Write (37%)<br>Read & write (26%)<br>Elementary (15%)<br>Secondary and above (22%)	36% of the participants perceived that they have a moderate risk of infection. with COVID-19<br><br>Half of the participants perceived the disease as highly threatening.<br><br>37% of study participants had good general knowledge of COVID-19.<br><br>Rural residents were nineteen times more likely to have poor knowledge and practice than urban residents.<br><br>Only 26% of study participants adopted good practices.

Appendix 2 – Overview of the main characteristics and findings of the studies included in the scoping review.

Authors	Title	Country	Target population	Survey administration	Sampling scheme	Sample size	Sample characteristics	Main findings
Alobuia WM, Dalva-Baird NP, et al.	Racial disparities in knowledge, attitudes and practices related to COVID-19 in the USA	United States	Adults living in the United States	Telephone interview	Random digit dial strategy	2906	Female (52%)<br>Male (48%)<br><br>18-29 (22%)<br>30-49 (34%)<br>50-64 (24%)<br>≥65 (20%)<br><br>Less than high school (9%)<br>High school (29%)<br>College and higher (61%)	63% worried about getting sick or a family member.<br><br>59% of respondents had good knowledge.<br><br>Black and Hispanic less likely than white to have high knowledge scores.<br><br>67% of respondents had good preventive practices.<br>Black and Hispanic were more likely to have high practice scores. Females more likely to have high practice scores compared to males.
Alsan M, Stantcheva et al.	Disparities in Coronavirus 2019 Reported Incidence, Knowledge, and Behavior Among US Adults	United States	Adults living in the United States	Online survey	Convenience	5198	Female (55%)<br>Male (45%)<br><br>18-29 (20%)<br>30-49 (30%)<br>50-59 (16%)<br>60-69 (18%)<br><br>College degree (51%)	Low/medium perceived risk<br><br>Knowledge about COVID-19 was high, but African American, men and younger people had less accurate knowledge than white respondents.<br><br>Preventive behaviors were generally high but African American, men and younger people were more likely to leave their homes
Austrian K, Pinchoff J et al.	COVID-19 related knowledge, attitudes, practices and needs of	Kenya	Households living in urban	Online survey	Random stratified sampling	2009	Female (62.7%)<br>Male (37.3%)	35% of respondents perceived that they were at high risk of COVID-19 infection. This

Appendix 2 – Overview of the main characteristics and findings of the studies included in the scoping review.

Authors	Title	Country	Target population	Survey administration	Sampling scheme	Sample size	Sample characteristics	Main findings
	households in informal settlements in Nairobi, Kenya		slums in Nairobi				18-24 (21.6%)<br>25-44 (54.6%)<br>≥45 (23.7%)<br><br>No School (3.6%)<br>Primary (39.6%)<br>Secondary (44%)<br>Higher (12.8%)	perception of risk increases by age groups.<br><br>Awareness of fever and dry cough as the top two symptoms of COVID-19 was high. Awareness was higher with increasing education.<br><br>Most respondents adopted appropriate preventive behaviors (97% washed their hands more frequently and 94% stopped attend social gatherings.
Banda J, Dube AN, et al.	Knowledge, risk perceptions and behaviors related to the COVID–19 pandemic in Malawi	Malawi	Adults living in Malawi	Telephone interview	Simple random and random stratified sampling	630	*Rural*<br>Women (59.0%)<br>Men (41%)<br><br>18–24 (19.2)<br>25–44 (65.2%)<br>45–54 (12.7)<br>≥55 (2.9)<br><br>*Urban*<br>Women (57.1)<br>Men (42.9%)<br><br>18–24 (19.3)<br>25–44 (66.4%)<br>45-54 (11.4)<br>≥55 (2.9)	Slightly less than half of the respondents perceived themselves to be at no risk or at low risk of infection.<br><br>3 out of four respondents expected to experience “severe” or “life threatening” symptoms.<br><br>In rural areas, only 5.3% of respondents reported being “almost certain” to become infected, vs. 12.1% in urban areas.<br><br>Respondents were moderately knowledgeable about the

Appendix 2 – Overview of the main characteristics and findings of the studies included in the scoping review.

Authors	Title	Country	Target population	Survey administration	Sampling scheme	Sample size	Sample characteristics	Main findings
								transmission patterns of the virus, and urban respondents were more aware of transmission patterns than rural respondents.<br><br>More than 95% of respondents reported washing their hands more frequently, and approximately 50% reported avoiding crowds.<br><br>The use of face masks and hand sanitizers was more prevalent among urban residents than in rural areas.
Bostan S, Erdem R, et al.	The Effect of COVID-19 Pandemic on the Turkish Society	Turkey	Adults living in Turkey	Online survey	Convenience	1586	Women (50.6%)<br>Men (49.4%)<br><br>≤29 (46.8%)<br>30-49 (40.3%)<br>50-59 (10.2%)<br>≥60 (2.3%)<br><br>Primary or secondary (2%)<br>High School (8.7%)<br>University (89.4%)	74% feel concern about transmitting COVID-19.<br><br>80% considered having enough knowledge about COVID-19.<br><br>Preventive practices generally high (97% of declared complying with general and hand hygiene rules)<br>Women more likely to adopt protective behaviors.
Bowman LR, Kwok KO, et al.	Public perceptions and preventive behaviours during the early phase of the COVID-19 1 pandemic: a comparative study	Hong-Kong and United-Kingdom	Adults living in Hong-Kong and adults living in the United-Kingdom	Online survey	Quota sampling	HK: 1663<br>UK: 1468	Women: 68.6% [HK] vs. 52.9% [UK]<br><br>18-24: 26.0% [HK] vs. 9.4% [UK]	Perceived severity differed by region (HK: 97%; UK: 21%)<br><br>A large proportion of respondents regarded direct

Appendix 2 – Overview of the main characteristics and findings of the studies included in the scoping review.

Authors	Title	Country	Target population	Survey administration	Sampling scheme	Sample size	Sample characteristics	Main findings
	between Hong Kong and the United Kingdom						25-44: 54.4% [HK] vs. 32.6% [UK]<br>45-54: 11.6% [HK] vs. 17% [UK]<br>≥55: % 7.9% [HK] vs. 41% [UK]<br><br>Lower secondary or below: 3.2% [HK] vs. 5.7% [UK]<br><br>Secondary level: 17.6% [HK] vs. 41.7% [UK]<br><br>Post-secondary: 16.1% [HK] vs. 18.9% [UK]<br><br>University degree level: 63.2% [HK] vs. 33.7% [UK]	contact with infected individuals as the transmission route of COVID-19 (HK: 94-98%; UK:69-93%), with HK respondents identifying other routes (wild animal meat, wet markets and imported goods)<br><br>Hand hygiene compliance was high for the HK (95%) and UK (91%) respondents<br><br>HK respondents reported higher levels of adoption of social distancing (avoiding crowded areas: HK: 87% vs. UK: 59%) and mask wearing (HK: 99%; UK: 3%).<br><br>General measures were less likely to be adopted by males. UK respondents were significantly less likely than their HK counterparts to adopt avoidance behaviors (e.g., avoiding crowded places)
Chan EYY, Huang Z, et al.	Sociodemographic Predictors of Health Risk Perception, Attitude and Behavior Practices Associated with Health-	Hong-Kong	Adults living in Hong-Kong	Telephone interview	Random digit dialing	765	Female (53.5%)<br>Male (46.5%)<br><br>18-24 (9.3%)<br>25-44 (32.4%)<br>45-64 (39.6%)	66,7% worried of being infected.<br><br>High level of knowledge (99% could identify that the disease could be transmitted through droplets)

Appendix 2 – Overview of the main characteristics and findings of the studies included in the scoping review.

Authors	Title	Country	Target population	Survey administration	Sampling scheme	Sample size	Sample characteristics	Main findings
	Emergency Disaster Risk Management for Biological Hazards: The Case of COVID-19 Pandemic in Hong Kong, SAR China						≥65 (18.7%)<br><br>Primary (8%)<br>Secondary (43.3%)<br>University (48.7%)	High level of appropriate behaviors, expect for social distancing in public.<br><br>Elderly and people with low education had relatively poor knowledge and less likely to adopt preventive behaviors
Chen Y, Zhou R, et al.	Knowledge, attitudes, and practices toward COVID-19 among Chinese older adults? An Online Cross-Sectional Survey	China	Older Chinese residents aged 60 and above	Online survey	Convenience	1263	Female (55.2%)<br>Male (44.8%)<br><br>60-69 (54.1%)<br>70-79 (37.5%)<br>>80 (8.4%)<br><br>Primary or below (46.4%)<br>Middle School (26.1%)<br>High School (15.6%)<br>College or above (11.9)	90% of the elderly believed older people may suffer from more severe symptoms.<br><br>85% of respondents did not perceive that the chances for them to contract COVID-19 were high/very high.<br><br>87% of the elderly were knowledgeable of COVID-19.<br><br>81% of our participants had increased handwashing and face-mask wearing in public venues (82%). 86% of the elderly had reduced their visits to crowded places.<br><br>Rural dwellers, and those attended at primary school or below had lower scores of preventive practices

Appendix 2 – Overview of the main characteristics and findings of the studies included in the scoping review.

Authors	Title	Country	Target population	Survey administration	Sampling scheme	Sample size	Sample characteristics	Main findings
Cvetković VM, Nikolić N et al.	Preparedness and Preventive Behaviors for a Pandemic Disaster Caused by COVID-19 in Serbia	Serbia	Adults living in Serbia	Online survey	Snowball	975	Female (76.6%)<br>Male (23.4%)<br><br>18-28 (72.6%)<br>29-38 (12.9%)<br>39-48 (9.2%)<br>49-58 (5.2%)<br><br>Primary (0.3%)<br>High School and College (31.1%)<br>University (68.2%)	Likelihood of getting sick was moderate (mean score: 3/5).<br><br>High level of knowledge<br><br>Respondents reported significant behavioral changes in hand hygiene, and social distancing.<br>Strongest predictor of good knowledge was high educational level and being a woman. Age, gender, and educational level were also significantly predictive to adopting preventive behaviors.
Faasse K et Newby J.	Public perceptions of COVID-19 in Australia: perceived risk, knowledge, health-protective behaviours, and vaccine intentions	Australia	Australian adult residents	Online survey	Convenience	2174	Female (75.2%)<br>Male (23.1%)<br>Different identity, or prefer not to say (1.7%)<br><br>18–29 (22.5%) 30–49 (39.4%)<br>50–59 (22.4%)<br>≥60 (13.9%)<br><br>High school (24.6%)<br>Trade certificate, diploma, or advanced diploma (24.3%)	The overall perceived susceptibility to the virus was moderate. 74 % reported that they would experience mild or moderate symptoms<br><br>95% of respondents knew that fever or cough were the more common symptoms but only 56% knew about airborne transmission.<br><br>Hygiene behaviors, including handwashing, or using hand sanitizing gel were the most commonly reported behaviors (93%) whereas only 34% of the

Appendix 2 – Overview of the main characteristics and findings of the studies included in the scoping review.

Authors	Title	Country	Target population	Survey administration	Sampling scheme	Sample size	Sample characteristics	Main findings
							University degree (50.9%)	respondents wore a face mask when going out in public.<br><br>Female respondents reported engaging in more protective behaviors than their male counterparts. Youngest (18–29) were engaged in fewer behaviors than older respondents.<br>Non-Caucasian (Asian and Australian aboriginal) respondents reported more protective behaviors.
Hakeem AR, Padmanaban H, et al.	Awareness and Concerns Among Adult Liver Transplant Recipients in the Current Pandemic Caused by Novel Coronavirus (COVID-19): Strategies to Safeguard a High-risk Population	India	Adult liver transplant and recipients living in India	Online survey	Convenience	112	Female (19%)<br>Male (81%)<br><br>Median age: 53 (range 18-64)<br><br>No education (2%)<br>School level (24%)<br>University (74%)	A majority aware of their high-risk status (strongly agree 74% and agree 21%)<br><br>A majority knew common symptoms, such as fever (92%), but only 26% felt that COVID-19 can be spread by asymptomatic patients.<br><br>71% stayed at home, but only 20% minimized contact with family members and visitors
Hezima A, Aljafari A, et al.	Knowledge, attitudes, and practices of Sudanese residents towards COVID-19	Sudan	Adult Sudanese citizens	In-person interview and online survey	Convenience	812	Female (45.8%)<br>Male (54.2%)<br><br>18-25 (51.1%)<br>26-45 (33.6%)<br>46-55 (10.7%)	The vast majority (93%) believed that COVID-19 is a serious threat for public health.<br><br>Mean knowledge score was 7.03/9. Women were found to

Appendix 2 – Overview of the main characteristics and findings of the studies included in the scoping review.

Authors	Title	Country	Target population	Survey administration	Sampling scheme	Sample size	Sample characteristics	Main findings
							≥55 (4.6%)<br><br>No education (5.7%)<br>High school (22.5%)<br>University (71.8%)	be more knowledgeable than male. People aged 18-25 were more knowledgeable than other age groups.<br><br>86% of respondents washed their hand frequently, 65% avoided crowded places, but only 34% wore masks.<br><br>Being a woman was significantly associated with wearing masks and avoiding crowded places. Being over 55 years was significantly associated with hand washing.
Ko N-Y, Lu W-H, et al.	Cognitive, Affective, and Behavioral Constructs of COVID-19 Health Beliefs: A Comparison Between Sexual Minority and Heterosexual Individuals in Taiwan	Taiwan	Heterosexuals and sexual minority adults living in Taiwan	Online survey	Convenience	1954	*Heterosexuals*<br>Female (72.2%)<br>Male (27.8%)<br>Mean age: 39.9<br>High School (12.7%)<br>University (87.3%)<br><br>*Sexual Minorities*<br>Female (52.3%)<br>Male (47.7%)<br>Mean age: 32<br>High School (6.9%)<br>University (93.1%)	Perceived susceptibility to COVID-19 was high in both groups (75%), but sexual minority participants were less worried about being infected than heterosexuals.<br><br>Perceived level of knowledge was high in both groups.<br><br>Avoiding crowded places was relatively high in both groups (83 % heterosexuals, 75% sexual minorities).

Appendix 2 – Overview of the main characteristics and findings of the studies included in the scoping review.

Authors	Title	Country	Target population	Survey administration	Sampling scheme	Sample size	Sample characteristics	Main findings
Lau LL, Hung N, et al.	Knowledge, attitudes, and practices of COVID-19 among income-poor households in the Philippines: A cross-sectional study	Philip-pines	Households experiencing extreme poverty in Philippines	In-person interview	Convenience	2224	Female (92.7%)<br>Male (7.3%)<br><br>≤20 (2.2%)<br>20-39 (48.6%)<br>40-59 (35.2%)<br>≥60 (14%)<br><br>No education (2.2%)<br>Primary (41.8%)<br>High School (46.4%)<br>College or higher (2.3%)	Most participants worried about contracting COVID-19 (80%)<br><br>89% were able to identify coughing and sneezing as a transmission route, but only 32% knew about social distancing and 49% about wearing face masks<br><br>Higher level of education was associated with greater knowledge of COVID-19 transmission routes<br><br>More than 60% reported avoiding crowded places, and keeping distance from people who were sick, but only 28% reported wearing a mask
Lee M, You M	Psychological and Behavioral Responses in South Korea During the Early Stages of Coronavirus Disease 2019 (COVID-19)	South Korea	Adult Korean residents	Online survey	Proportionat e quota	973	Female (50.1%)<br>Male (49.9%)<br><br>18-29 (17.7%)<br>30-49 (36.8%)<br>50-59 (20.5%)<br>≥60 (25.1%)<br><br>Under high school (53.4%)<br>College or above (46.6%)	Respondents had a low-moderate perceived susceptibility to COVID-19.<br><br>Age and perceived health were negatively associated with perceived susceptibility and severity.<br><br>28% of the respondents reported that their knowledge was low or very low.

Appendix 2 – Overview of the main characteristics and findings of the studies included in the scoping review.

Authors	Title	Country	Target population	Survey administration	Sampling scheme	Sample size	Sample characteristics	Main findings
								The most frequently practiced precautionary behavior was hand hygiene. 63% reported always wearing a facial mask when outside, but only 39 % reported they reduced using public transports.<br><br>Gender, education level were positive and significant individual predictors of wearing facial masks.
Leigh JP, Fiest K, et al.	A national cross-sectional survey of public perceptions, knowledge, and behaviors during the COVID-19 pandemic	Canada	Adults residing in Canada	Online	Quota sampling	1,996	Women (54.3%), men (45.2%)<br>Other (0.5%)<br><br>18-29 (15.2%)<br>30-44 (25.3%)<br>45-64 (31.9%)<br>≥65 (27.6%)<br><br>High school or less (20.1%)<br>Technical college/some college (24.1%)<br>College/University degree (55.9%)	More respondents were moderately or extremely concerned about a family member contracting COVID-19 (45%) but less worried about contracting themselves the virus (30%)<br><br>Respondents from more populated provinces (Ontario and Quebec) were more concerned than those in populated provinces about contracting COVID-19.<br><br>Most respondents (88%) felt they had good to excellent knowledge of virus transmission and were very knowledgeable regarding asymptomatic transmission (86%).

Appendix 2 – Overview of the main characteristics and findings of the studies included in the scoping review.

Authors	Title	Country	Target population	Survey administration	Sampling scheme	Sample size	Sample characteristics	Main findings
								Respondents reported being highly compliant with distancing measures (95% always or often practicing physical distancing) and 43% practiced self-isolation.
Malik AA, McFadden S, et al.	COVID-19 Risk Perception Among U.S. Adults: Changes from February to May 2020	United States	United States adult population	Online survey	Quota sampling	672	18-25 (11%)<br>26-45 (37%)<br>46-55 (14%)<br>≥55 (38%)<br>No high School (2%)<br>High School (24%)<br>Some college/ College (55%) Graduate/ Professional (22%)	The average risk perception score was moderate (5.9/10). When compared to results from our February 2020 survey, participants from the May survey perceived they were at higher risk of contracting COVID-19<br><br>Adults over 55 years of age reported higher risk perception than younger adults. American/Alaska Native and Asian respondents also reported increased risk perception compared to participants who reported being Black/African American<br><br>72% of our sample said they had good or very good knowledge about COVID-19 (compared to 39% of respondents in February)<br><br>Over 90% of respondents reported following CDC

Appendix 2 – Overview of the main characteristics and findings of the studies included in the scoping review.

Authors	Title	Country	Target population	Survey administration	Sampling scheme	Sample size	Sample characteristics	Main findings
								guidelines, and 85% used a face mask.
Pagnini F, Bonanomi A, et al.	Knowledge, Concerns, and Behaviors of Individuals During the First Week of the Coronavirus Disease 2019 Pandemic in Italy	Italy	People living in Italy in different areas (hot spots and less affected areas)	Online survey	Snowball sampling	2286	Mean age (SD): 30.7 (13.2) years women (76 %) Men (24%) Primary school (0.1%) Middle school (3%) High school diploma (46%) University degree (51%)	The mean score of perceived susceptibility was moderate 3.8/5<br><br>In hot spots and less affected areas, scores for worries were higher among women than men. Older participants were less worried about their risk of getting sick. People living in the green zones had lower mean scores for worries about getting sick (4.2/5 in red zones vs.3.57/5 in green zones)<br><br>77 % of respondents had a good knowledge of COVID-19.<br><br>People in red zones had higher mean scores for preventive behaviors than people living in the other zones (4.25/5 in red zones vs. 2.88/5 in green zones).
Pal R, Yadav U et al.	Knowledge, attitudes, and practices towards COVID-19 among young adults with Type 1 Diabetes Mellitus amid the nationwide lockdown in	India	Young adults with type 1 diabetes mellitus	Online survey	Convenience	212	Age (mean ± SD) 25.1 ± 4.3 years<br><br>Men (48%)<br>Women (52%)	88% of the respondents though as a patient with diabetes mellitus they were at a higher risk of getting infected.<br><br>75% felt that they were more at risk of complications.

Appendix 2 – Overview of the main characteristics and findings of the studies included in the scoping review.

Authors	Title	Country	Target population	Survey administration	Sampling scheme	Sample size	Sample characteristics	Main findings
	India: A cross-sectional survey						Educated up to 10th standard (9%)<br>Educated up to 12th standard 39 (18%)<br>Graduate (41%)<br>Post-graduate (32%)	The mean total knowledge score was 12.4/15 (overall correct rate of 83%).<br><br>Higher educational status, and urban residence were associated with better knowledge scores<br><br>100% of the respondents reported wearing a mask and washing their hands more frequently.
Parikh PA, Shah BV, et al.	COVID-19 Pandemic: Knowledge and Perceptions of the Public and Healthcare Professionals	India	Adult Indian residents	Online survey	Convenience	1246	Female (43.6%)<br>Male (56.4%)<br><br>Mean age: 32	82% of the respondents were worried about getting COVID-19.<br><br>Respondents were very knowledgeable about COVID-19 (symptoms, routes of transmission and preventive behaviors).<br><br>Respondents were quite well informed about COVID-19 and aware of the measures needed to be taken.
Paul A, Sikdar D, et al.	Knowledge, attitude and practice towards the novel corona virus among Bangladeshi 2 people:	Bangla-desh	Adult Bangladeshi population	Online survey	Snowball	1589	Female (39.5%)<br>Male (60.5%)<br><br>18-25 (46.5%)<br>26-45 (45.1%)	55 % of the respondents perceived COVID-19 as a deadly disease, curable with low mortality rate

Appendix 2 – Overview of the main characteristics and findings of the studies included in the scoping review.

Authors	Title	Country	Target population	Survey administration	Sampling scheme	Sample size	Sample characteristics	Main findings
	Implications for mitigation measures						46-65 (7.8%)<br>≥65 (0.6%)<br><br>Secondary and below (4.2%)<br>University (95.8%)	Respondents demonstrated poor knowledge scores. Older people were more knowledgeable than younger. 80 % of respondents avoided crowded places, 79% used a mask.
Reuken PA, Rauchfuss F, Albers S	Between fear and courage: Attitudes, beliefs, and behavior of liver transplantation recipients and waiting list candidates during the COVID-19 pandemic	Germany	Adults recipients and candidates for liver transplant in Germany and household members	Mail	Convenience	871	*Organ transplant recipients*<br>Female (38%)<br>Male (62%)<br>Mean age: 62<br>Secondary (64%)<br>High School (8%)<br>University (28%)<br><br>*Organ transplant candidates*<br>Female (65%)<br>Male (35%)<br>Mean age: 59<br>Secondary (72%)<br>High School (6%)<br>University (21%)	Organ transplant recipients (64%) and organ transplant candidates (54%) were afraid to become infected.<br><br>Female sex was significantly associated with fear of being infected with SARS-CoV-2.<br><br>Most patients felt informed about COVID-19 (80%).<br><br>72% of organ transplant candidates were leaving the house less frequently and most patients reported wearing gloves and/or face mask when leaving the house.
Roy D, Tripathy S, et al.	Study of knowledge, attitude, anxiety & perceived mental healthcare need in Indian population during COVID-19 pandemic	India	Adult Indian citizens	Online survey	Snowball	662	Female (51%)<br>Male (49%)<br>Mean age: 29	72 % of participants reported being worried to be infected.<br><br>Respondents were moderately aware of the basic elements of the disease. Only 18% regarded fever as a symptom of COVID-19.

Appendix 2 – Overview of the main characteristics and findings of the studies included in the scoping review.

Authors	Title	Country	Target population	Survey administration	Sampling scheme	Sample size	Sample characteristics	Main findings
								82 % of the respondents had reduced social contact, but only 37 % of participants wore a mask.
Saikarthik J, Saraswathi I, et al.	Risk factors and protective factors of mental health during COVID-19 outbreak and lockdown in adult Indian population- A cross-sectional study	India	Adult Indian residents	Online survey	Snowball sampling	873	Female (45.9%)<br>Male (54.1%)<br><br>18-25 (31.6%)<br>26-35 (22.7%)<br>36-45 (30.8%)<br>46-55 (4.5%)<br>≥56 (7.2%)<br><br>No education (3.2%)<br>Higher secondary (29.8%)<br>University (66.8%)	61% of respondents reported to be highly likely or likely to contract COVID-19.<br><br>33% reported to be not highly likely or not likely to survive from COVID-19.<br><br>71% of the respondents knew about transmission through droplets and only 23% about airborne transmission.<br><br>71% of the respondents reported washing their hands more than 5 times a day<br><br>74% reported wearing a face mask while being outside in public places.
Ssebuufu R, Sikakulya FK et al	Awareness, knowledge, attitude, and practice towards measures for prevention of the spread of COVID-19 in the Ugandans: A nationwide online cross-sectional Survey	Uganda	Adult Ugandan residents	Online survey	Snowball	1763	Female (43.1%)<br>Male (56.9%)<br><br>18-30 (50.6%)<br>31-50 (44.2%)<br>>50 (5.2%)	97% of participants thought that COVID-19 can lead to death.<br><br>Most respondents were highly knowledgeable about COVID-19 (84%)

Appendix 2 – Overview of the main characteristics and findings of the studies included in the scoping review.

Authors	Title	Country	Target population	Survey administration	Sampling scheme	Sample size	Sample characteristics	Main findings
								Most respondents (85%) practiced the main preventive measures (hand washing, social distancing, mask wearing).
Wolf MS, Serper M et al	Awareness, Attitudes, and Actions Related to COVID-19 Among Adults With Chronic Conditions at the Onset of the U.S. Outbreak: A Cross-sectional Survey	United States	Adults living with one or more chronic condition in the United States	Telephone interview	Convenience	630	Female (59.7%)<br>Male (40.3%)<br><br>≤60 (37.3%)<br>60-69 (35.7%)<br>≥70 (27%)	25% of respondents said that they were "very worried" about getting COVID-19 and 13% were not worried at all.<br><br>Black participants were more likely than white participants to report that they were "not worried at all" about getting COVID-19.<br><br>Most participants correctly identified three symptoms (72%) and three ways to prevent the infection (70%).<br><br>Participants who were older and black showed poorer knowledge of COVID-19<br><br>More than half of patients (59%) reported that COVID-19 had caused them to change their daily routine "a lot"
Yassa M, Birol P et al	Near-term pregnant women s attitude toward, concern about and knowledge of the COVID-19 pandemic	Turkey	Non infected pregnant women near term living in Turkey	Paper survey	Convenience	172	Mean age: 27	51% feel more at risk than non-pregnant women. 35% constantly fear about being infected, 41% think that their

Appendix 2 – Overview of the main characteristics and findings of the studies included in the scoping review.

Authors	Title	Country	Target population	Survey administration	Sampling scheme	Sample size	Sample characteristics	Main findings
								baby could be infected after birth<br><br>50% of women thought that breastfeeding is safe, 76% of women did not know if COVID-19 might cause birth defects, and 64% did not know if COVID-19 might cause preterm births. 73% of women thought that they were taken adequate precautions.
Yıldırım M et Guler A	COVID-19 severity, self-efficacy, knowledge, preventive behaviors, and mental health in Turkey	Turkey	Turkish adults	Online survey	Convenience	3190	Women (50%), Men (50%)<br><br>Mean age: 39 years (SD :10.4)<br><br>High school and below (5.2%)<br>University (94,8%)	Respondents reported a high level of perceived severity (8.12/10)<br><br>64 % of the participants said they had never heard about COVID-19.<br><br>Wearing a mask (4,74/5), avoiding public transportation (4.72/5), and avoiding public gathering (4,65/5) were the most frequently practiced preventive behaviors
Zipprich HM, Teschner U et al	Knowledge, Attitudes, Practices, and Burden During the COVID-19 Pandemic in People with Parkinson's Disease in Germany	Germany	Patients with Parkinson disease living in Germany	Telephone interview	Convenience	99	*Older group*<br>Female (38.6%)<br>Male (61.4%)<br>Mean age: 78<br>Low education (22.8%)<br>Middle education (22.8%)	Most patients (95%) stated that the virus was dangerous.<br><br>Most patients reported that they were well or very well informed about COVID-19 (70%).

Appendix 2 – Overview of the main characteristics and findings of the studies included in the scoping review.

Authors	Title	Country	Target population	Survey administration	Sampling scheme	Sample size	Sample characteristics	Main findings
							High education (54.4%)<br><br>*Younger group*<br>Female (31%)<br>Male (69%)<br>Mean age: 65<br>Low education (14.3%)<br>Middle education (52.3%)<br>High education (33.3%)	Most patients (73%) reported that they had changed their behaviors since the appearance of COVID-19.<br><br>The most common actively reported preventive behaviors were staying at home (60%), reduced social contact (40%), wash hands (40%), wearing mask (40%).
